# Uncontrolled hypertension among hypertensive patients on treatment in Lupane District, Zimbabwe, 2012

**DOI:** 10.1186/1756-0500-7-703

**Published:** 2014-10-08

**Authors:** Tafadzwa Priscilla Goverwa, Nyasha Masuka, Mufuta Tshimanga, Notion Tafara Gombe, Lucia Takundwa, Donewell Bangure, Maureen Wellington

**Affiliations:** Department of Community Medicine, University of Zimbabwe, Harare, Zimbabwe; Provincial Medical Directorate, Matabeleland North Province (MoHCC), Matabeleland, Zimbabwe

**Keywords:** Uncontrolled, Hypertension, Lupane district, Zimbabwe

## Abstract

**Background:**

More than half of hypertensive patients reviewed at Lupane District Hospital during the first half of 2011 had uncontrolled hypertension. This prompted an investigation on the prevalence of uncontrolled hypertension and associated factors among hypertensives on treatment.

**Methods:**

Analytical cross-sectional study was conducted. Three hundred fifty-four consenting participants were consecutively selected from eligible hypertensive patients on treatment attending the outpatients department at Lupane District Hospital for their reviews. An interviewer administered questionnaire adapted from the World Health Organization was used to collect data on risk factors. Blood pressure and anthropometric measurements were taken as per World Health Organization guidelines. Uncontrolled hypertension was defined as systolic blood pressure of ≥140 mmHg and/or diastolic blood pressure of ≥90 mmHg in a patient taking anti-hypertensive medication.

**Results:**

Mean systolic BP was 151.0 mmHg and mean diastolic BP was 92.6 mmHg. Prevalence of uncontrolled hypertension was (238) 67.2%. Independent risk factors for uncontrolled hypertension were obesity (AOR 3.28, 95% CI 1.39-7.75) and adding salt to food at the table (AOR 2.77, 95% CI 1.41-5.43) whilst being compliant with the drug treatment regimen (AOR 0.34, 95% CI 0.16-0.72) and having received health education on hypertension (AOR 0.49, 95% CI 0.25- 0.97) were protective against uncontrolled hypertension.

**Conclusion:**

There prevalence of uncontrolled hypertension is high despite all the participants being on treatment. The findings suggest that interventions at the patient, the provider and the health delivery system are needed to improve hypertension control in Lupane, Zimbabwe.

## Background

Hypertension is a major public health problem associated with high morbidity and mortality rates globally [1]. It is the largest risk factor for cardiovascular diseases, growing in prevalence and poorly controlled virtually everywhere [2]. The treatment and control of hypertension can lead to a reduced incidence of complications, including stroke, coronary heart disease, heart failure, and kidney disease [2]. Despite this, most hypertensive patients have poorly controlled hypertension [3]. Recent studies also indicate that awareness and management of hypertension is suboptimal especially in the developing world [4–6].

Limitations in the extent of the control of hypertension in the population are commonly attributed to health service related factors and patient related factors. Health service related factors including financial and geographical accessibility of the available health facilities, the availability and clinical practice of clinicians together with the availability of essential medicines have been cited as important factors in the control of hypertension [7]. Patient related factors such as socio-economic status, educational level, obesity and behaviors such as excessive alcohol consumption and cigarette smoking have also been identified through studies as reasons for uncontrolled hypertension [7].

Zimbabwe like several other developing countries and in line with the so called epidemiological transition is faced with an increasing burden of non-communicable diseases (NCDs) and hypertension is the leading NCD in the country [8]. Hypertension accounts for 50% of all cardio-vascular diseases and its complications including strokes [6]. In the Zimbabwe STEPwise survey conducted in 2005 the prevalence of hypertension was found to be as high as 27 percent, while in 2006 hypertension accounted for 25% of all out- patient visits for chronic conditions [9]. In Matabeleland North province hypertension is the commonest chronic non-communicable disease and in 2011 an estimated 46% of the chronic NCDs among patients seen at OPD presented with hypertension.

Whilst there has been an attempt to determine the prevalence of hypertension in Zimbabwe, there is paucity of literature on the control rates of the disease among those on treatment. A preliminary analysis of the chronic disease register at Lupane District hospital indicated that more than half of the hypertensive patients on treatment had uncontrolled hypertension in 2011. In addition, the number of days spent in hospital by hypertensive patients admitted with complications due to hypertension was also high. The added burden of diseases as a result of complications from uncontrolled hypertension places additional pressure on the limited health care budget in Lupane district, Matebeleland North province and the nation at large. Thus adequate control of hypertension among hypertensive patients is of enormous public health importance. The aim of this study was to determine the prevalence of uncontrolled hypertension and to identify the underlying factors associated with uncontrolled hypertension amongst sufferers so as to develop effective interventions to overcome these factors.

## Methods

An analytical cross-sectional study was carried out at Lupane District Hospital outpatients department. The study population was consenting hypertensive patients on treatment, 18 years old and above and resident in Lupane district. Additional inclusion criteria for the analytic part of the study were having a diagnosis of hypertension for at least six months and being on the current anti-hypertensive medication regimen for at least two months prior to the study. All hypertensive patients who presented for their reviews were consecutively recruited into the study depending on their eligibility. A minimum sample size of 384 was required for the study.

Three seated blood pressure (BP) measurements at ≥2-minute intervals, after 5 minutes rest were obtained from each participant by the principal investigator assisted by a trained research assistant according to the WHO protocol [10]. The average BP of the last 2 measurements was used for the study and participants were designated as having uncontrolled hypertension if they had a systolic BP ≥140 mm Hg and/or a diastolic BP ≥90 mm Hg and were on antihypertensive medication. Body weight was measured to the nearest 0.1 kg using a digital scale and height to the nearest 0.1 cm in the standing position using a portable height board. Body mass index (BMI) was calculated as weight (in kilograms) divided by squared height (in meters squared) and further divided into the categories defined by the World Health Organization: <18.5 kg/m^2^ (lean), 18.5-24.9 kg/m^2^ (normal), 25.0-29.9 kg/m^2^ (over weight), and 30+ kg/m^2^ (obese) [11].

A structured interviewer administered questionnaire adapted from the Zimbabwe STEPwise survey [9] and translated into the local language (Ndebele) was administered to solicit information on socio-demographic, lifestyles and other variables. The modified 10-item Hill Bone Compliance scale was used to measure self-reported compliance to anti-hypertensive medication [12]. A 9-item scale adopted from a study conducted in Seychelles on factors affecting drug treatment compliance among hypertensives in Praslin Island [13] was used to measure the perceived risk of complications due to hypertension. This measured the respondent’s perceived probability of developing complications from hypertension. Physical activity was assessed by asking the participants the number of minutes per day and the number of days per week spent doing vigorous, moderate and light activities. Metabolic Equivalents (METS) were calculated by multiplying the number of minutes spent on vigorous activities by 8.0, moderate activities by 4.0 and light activities by 1.0. Using the total number of METS per week participants were classified into physically active and physically inactive.

A checklist was used to collect information from the participant’s medical records on the treatment regimen the individual was on, blood pressure control on the previous 3 visits, the presence of co-morbid conditions. Statistical analyses were performed using the Statistical Package for Social Sciences (SPSS) to generate frequencies, proportions, tables, graphs, perform *X*^2^ test of association, and calculate odds ratios, confidence intervals and p-values. Ethical clearance was obtained from the Joint Review Ethical committee for Parirenyatwa Hospital and University of Zimbabwe College of Health Sciences (JREC) and Medical Research Council of Zimbabwe (MRCZ). Informed written consent was obtained from the study participants.

## Results

Three hundred and fifty-four participants were interviewed and Table 1 shows their socio-demographic characteristics. The majority (62.7%) was females and was above 65 years of age (46.1%). The median age was 63.0 years (Q_1_ = 52.0, Q_3_ = 74.0).

**Table 1:** **Frequency distribution of socio-demographic characteristics of hypertensive patients on treatment in Lupane District, Zimbabwe, 2012**

Variable		Frequency	
	Males n = 132 (%)	Females n = 222 (%)	Overall n = 354(%)
**Marital status**			
Married	80 (22.6)	108 (30.5)	188 (53.1)
Single	6 (2.0)	20 (5.4)	26 (7.4)
Separated/Divorced	8 (2.3)	18 (5.1)	26 (7.4)
Widowed	38 (11.0)	76 (21.1)	114 (32.1)
**Highest level of education attained**			
None	6 (1.7)	22 (6.2)	28 (7.9)
Primary	44 (12.4)	138 (39.0)	182 (51.4)
Secondary	68 (19.2)	54 (15.3)	122 (34.5)
Tertiary	14 (4.0)	8 (2.3)	22 (6.2)
**Occupation**			
Employed	28 (7.9)	29 (8.2)	57 (16.1)
Unemployed	78 (22.0)	187 (52.9)	265 (74.9)
Retired	26 (7.3)	6 (1.7)	32 (9.0)
**Average monthly household income**			
<US$ 50	78 (22.0)	154 (43.5)	232 (65.5)
US$ 51-US$ 100	26 (7.3)	35 (9.9)	61 (17.2)
US$ 101-US$ 200	12 (3.3)	16 (4.6)	28 (7.9)
>US$ 200	16 (4.5)	17 (4.9)	33 (9.4)

**Figure 1:**
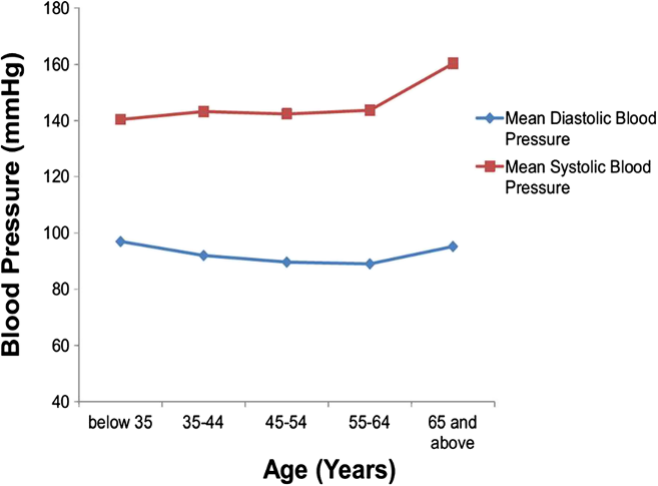
**Distribution of mean systolic and mean diastolic blood pressure with age group among hypertensive patients on treatment in Lupane district, 2012.**

The overall mean systolic blood pressure (SBP) was 151.0 ± 24.6 mmHg with mean of 151.4 ± 23.5 mmHg and 150.7 ± 25.3 mmHg among males and females respectively. The overall mean diastolic blood pressure was 92.6 ± 13.9 mmHg with mean of 93.1 ± 16.0 and 92.3 ± 12.3 mmHg among males and females respectively. The mean SBP increased with age (Figure 1). The mean body mass index (BMI) was 25.4 ± 4.06 kg/m^2^ among males and 28.3 ± 6.12 kg/m^2^ among females and 27.2 ± 5.62 kg/m^2^ overally. Two hundred and twenty-two (62.7%) had an abnormal BMI and the overall prevalence of obesity was 25.5% which was more prevalent among females (34.2%) compared to males (10.6%).

Two hundred and thirty-eight (67.2%) participants had uncontrolled hypertension and there were no significant gender differences (p = 0.08). The participant’s medical records revealed that 226 (63.8%) had a history of uncontrolled blood pressure in at least two out of the last three visits for their hypertension. Uncontrolled hypertension was also found to be higher in the older age groups with more than half (52.9%) of the cases being 65 years and above.

One hundred and eighty-nine (53.1%) had been diagnosed of hypertension more than five years before the study. All were on anti-hypertensive medication and most (52.6%) had been on treatment for more than five years before the study. Two hundred and thirty-three (65.9%) were taking one type of medicine, 99 (28%) were taking two medicines and only 22 (6.2%) were taking three or more medicines for their hypertension. More than half (54.8%) were on single dose regimen whilst (45.2%) were on multiple dose regimens. Two hundred and twenty (62.1%) participants were getting their medicines from the health facility for free, 128 (36.2%) were buying the medicines at the health facility and only 6 (1.7%) were buying from private pharmacies. Those buying from private pharmacies reported that the medicines which they are taking are not usually stocked at the health facility pharmacy. Fifty-eight percent (204) of the participants indicated that the medicines they are taking for hypertension were available throughout the previous 12 months of the study. Most (54.8%) of those who failed to get medicine from the health facility at least once in the preceding 12 months of the study due to stock-outs waited until the medicines were available because they could not afford to buy from private pharmacies.

Two hundred and fifteen (60.7%) had received health education on hypertension and 223 (63%) had been advised by their healthcare provider to reduce salt intake, 220 (62.1%) had been advised to engage in physical exercise or to lose weight. One hundred and eighty-four (54.0%) indicated that they had taken traditional herbs for the treatment of their hypertension and 58 (16.4%) reported having consulted a traditional/faith healer for their hypertension in the preceding 12 months of the study.

From the participants’ medical records, 182 (51.4%) had written evidence of co-morbidity and diabetes mellitus was the most common (37.4%) co-morbid condition followed by arthritis (32.9%). More than half (54.3%) had not taken their morning dose of medicine on the morning of the visit and more than half (52.8%) had missed at least one clinic visit in the preceding six months of the study. The Cronbach’s alpha for the 10-item compliance scale was 0.947. The overall mean score on the scale was 16.71 ± 6.00 with a mean score of 18.2 ± 6.50 for males and 15.8 ± 5.52 for females implying females were more compliant to the drug treatment regimen than males.

The cronbach’s alpha for the 9-item scale used to measure one’s perception of risk of developing complications as a result of hypertension was 0.935. The respondents perceived themselves to be most at risk of developing paralysis (74.3%), heart problems(68.6%) and stroke (69.5%) whilst they perceived themselves to be least at risk of developing visual impairment (42.1%) and having their careers negatively affected (49.7%).

Fifty-seven (16.1%) were current tobacco smokers with males being three times more likely to be smokers compared to females. Fifty-two (14.7%) were former smokers whilst 233 (65.8%) had never smoked. One hundred and twenty-eight (36.2%) had ever consumed an alcoholic drink and 54 (15.3%) had consumed alcohol in the previous three months of the study. Of these, 8 (14.8%) had taken alcohol at least 5 days per week, 30 (55.6%) had taken alcohol between 1–4 days per week, 12 (22.2%) between1-3days per month and only 4 (7.4%) had consumed alcohol less than once per month. Two hundred and six (58.2%) participants reported adding salt to their food at the table and this was more common among the males (72.7%) than females (49.5%). Most (95.5%) reported that their meals are usually prepared at home. Fruits consumption was low with the median number of days on which one has at least one serving of fruit in a typical week being 0.0 days (Q1 = 0.0, Q3 = 2.00). In contrast, vegetable consumption was found to be higher. The median number of days on which one has at least one serving of vegetables in a typical week was 5.0 days (Q1 = 5.00, Q3 = 7.00 days).

The overall prevalence of physical inactivity was 37.3%, with 48.5% in females and 30.6% in males. Most participants (75.7%) walked or cycled at least 10 minutes to and from places daily. Two hundred and ten (59.3%) were involved in everyday work activities of moderate intensity and females were more than twice (43.1%) more active in these work activities than males (15.2%). Only 6 (1.7%) were involved in vigorous and moderate intensity sport and all were males.

Out of the 354 study participants 334 (94.4%) were included in the analysis for factors associated with uncontrolled hypertension. The excluded 20 participants had been on treatment for hypertension for less than 6 months. Being 65 years of age and above was more than twice (OR 2.37, CI 1.47-3.82) associated with uncontrolled hypertension compared to those younger than 65 years of age. Other socio-demographic factors which were risk factors for uncontrolled hypertension were education level of primary and below and being female although not statistically significant. Those who were married, employed and had an average monthly household income above US$200 were less likely to have uncontrolled hypertension (Table 2).

**Table 2:** **Socio-demographic factors associated with uncontrolled hypertension among hypertensive patients on treatment in Lupane District, Zimbabwe 2012**

Factors		Uncontrolled	Controlled	Odds ratio	95% confidence interval
**Age**	65 years and above	120	35	2.37	1.47- 3.82*
	Less than 65 years	104	75		
**Education level**	Primary	143	62	1.34	0.84 – 2.13
	Secondary/+	81	48		
**Marital status**	Married	102	71	0.44	0.28- 0.71*
	Not married	122	39		
**Sex**	Female	141	68	0.97	0.61-1.56
	Male	83	42		
**Occupation**	Employed	28	24	0.51	0.28 – 0.93
	Unemployed	196	86		
**Income**	≥US$ 200	11	13	0.38	0.223 - 0.82*
	<US$ 200	213	97		
**Transport fare to health facility for review**	Nothing	109	57	0.89	0.78- 1.219
	US$1 and more	115	53		

*Statistically significant.

The lifestyle related risk factors for uncontrolled hypertension were adding salt to food at the table (OR 5.56, CI 3.01-8.040) currently smoking (OR 3.85, CI 1.75-9.20) and alcohol consumption (OR 1.94 CI 1.56-4.35). Physical inactivity (OR 1.03, CI 0.87 - 4.12) was not significantly associated with uncontrolled hypertension. Those who were on a single dose medication regimen were 65% less likely (OR 0.35, CI (0.21 - 0.60) to have uncontrolled hypertension compared to those on multiple dose regime. Visiting a traditional healer in the past 12 months (OR 3.47, CI (1.58-7.64) and taking traditional herbs in the past 12 months (OR 1.60, CI 1.01-2.53) were risk factors for uncontrolled hypertension. Co-morbidity in general was not significantly associated with uncontrolled hypertension (OR 1.15, CI 0.77-1.88). However being diabetic (OR 1.98, CI 1.04-3.75) and obese (OR 2.98, CI 1.59-5.62) were associated with uncontrolled hypertension. There was no statistically significant association between having been diagnosed of hypertension for more than five years (OR 1.54, CI 0.98-2.44), being on treatment for more than five years (OR 0.89, CI 0.68-1.06) and having one’s blood pressure measured at least once a month (OR 0.68, CI 0.44-1.02).

The mean score on the compliance scale for those with controlled and uncontrolled hypertension were 12.16 (95% CI 11.57- 12.74) and 18.93 (95% CI 18.19- 19.67) respectively. The difference between the two groups was statistically significant (p = <0.001) implying those with uncontrolled hypertension were less likely to be compliant compared to those with controlled hypertension. On a categorical scale being compliant (those with a total compliance score of 10) was found to be protective against uncontrolled hypertension (OR 0.15, CI 0.09-0.25). Those who took their morning dose of medicine on the day of the visit were 43% less likely to have uncontrolled hypertension (OR 0.57, CI 0.22-0.96).

Participants with a high risk perception (mean score of 3 and above) were almost 86% (OR = 0.14, 95% CI 0.084; 0,235) less likely to have uncontrolled hypertension compared to those whose perception was low. Those who had received health education on hypertension were 55% less likely to have uncontrolled hypertension compared to those who had not been educated on hypertension (OR 0.45, 95% CI 0.23-0.89).

After multivariate analysis independent factors associated with uncontrolled hypertension were being obese (AOR 3.28, 95% CI 1.39; 7.75), adding salt to food at the table (AOR 2.77, 95% CI 1.41; 5.43), being compliant with the drug treatment regimen (AOR 0.34, 95% CI 0.16; 0.72), having received health education on hypertension (AOR 0.49, 95% CI 0.25; 0.97) and having a high perception of the risk for developing complications due to hypertension (AOR 0.40, 95% CI 0.20; 0.84).

## Discussion

The prevalence of uncontrolled hypertension among hypertensive patients on treatment in Lupane was high (62.7%) and the recurrence of uncontrolled hypertension in almost two thirds of the participants in at least two of the previous three visits also implies that uncontrolled hypertension is a perennial problem. This is consistent with what has been found from other studies in developing and developed countries, for example in a study in South Africa on hypertension care and control among blacks, 39.9% on treatment for hypertension was adequately controlled [14]. Another study in Cuba demonstrated that the level of hypertension control among treated hypertensives was 39.4% among blacks [15]. Hypertension is the largest risk factor for cardiovascular diseases and treatment and control of hypertension can lead to a reduced incidence of complications [2]. Thus strategies for control of hypertension are required to reduce the burden of disease as a result of complications of uncontrolled hypertension in Lupane.

Age was associated with uncontrolled hypertension supporting evidence from literature that age is most strongly related to systolic blood pressure and isolated systolic hypertension accounts for the majority of cases with uncontrolled hypertension in individuals greater than 60 years of age [16]. However according to the Zimbabwean hypertension treatment guidelines, the systolic threshold for initiating therapy in the elderly is 160 mmHg and this is higher than the threshold for uncontrolled blood pressure [17]. This could account for the high prevalence of uncontrolled hypertension in this age group. Elderly patients may also be at risk of non-compliance with their medications due to forgetfulness thus leading to uncontrolled hypertension. Based on this result, interventions targeting elderly hypertensives should be initiated to reduce cardiovascular risks associated with uncontrolled hypertension in this population.

In this study, there were no statistically significant differences with respect to the relationship between gender and uncontrolled hypertension. It is important to note that literature on the association of gender with hypertension control has not been consistent. In the NHANES III (1988–1994) the rates of awareness and control were found to be higher in women compared with men [7]. A few years later in the 1999 and 2000 NHANES no significant gender differences were found with respect to hypertension control [7]. Other studies have reported no differences whilst others have reported better control in men [18, 19].

The association between obesity and uncontrolled hypertension found in this study has also been demonstrated in previous studies [20, 14]. Obese patients on anti-hypertensive medications are less likely to reach the recommended targets than their normal weight counterparts [21]. Studies have also shown that modest weight losses will not only reverse blood pressure elevations but will also have a favorable impact on obesity related cardiovascular risk factors such as diabetes and lipidaemias [21]. Co-morbidity in general was not statistically associated with uncontrolled hypertension but those who were diabetic were at risk of having uncontrolled hypertension. Thus among those with co-morbid conditions special attention is required for those with hypertension co-existing with diabetes mellitus since poor control of both conditions increases their risk for cardiovascular complications [20].

The proportion of participants who usually add salt to food at the table (58.3%) was not appreciably different from those who had been advised to reduce their salt intake. This may be an indication of low levels of compliance with lifestyle modifications or that the message is not well tailored to suit the audience. It is also important to note that, it takes about 8–12 weeks for the taste buds to become accustomed to low salt intake in someone who was previously used to high or moderate salt intake [22]. This therefore means that the individual has to be highly motivated and consistent.

Those with an average monthly household income of at least US$200 and the employed were less likely to have uncontrolled hypertension whilst low education level was as a risk factor. Those employed are more likely to be better educated with higher incomes and more knowledgeable on their hypertension and its control. The contribution of lower socio-economic status to hypertension control has also been demonstrated in other studies [18, 23]. However there was no association with uncontrolled hypertension among those who get their medication for free from the health facility and those who buy. This could be because most of those who buy the medication were buying from the health facility were the prices are subsidized and hence much cheaper than the private pharmacies. In addition at the health facility, no one is turned away if they have no money as the medicines can be given on credit.

Compliance with treatment has been widely recognized as key in achieving blood pressure control [13, 14] ^.^ We found that compliance with treatment and clinic visits were strongly associated with uncontrolled hypertension. Reasons for poor compliance may be insufficient patient knowledge, inaccurate perception about hypertension and medication costs and availability [7]. More than two-fifths of the participants reported not taking their medication on the morning of the visit. This could have resulted from some patients running out of their medicines a few days before the visit or the patients leaving their homes too early before they have eaten anything against the general belief that medicines should not be taken on an empty stomach.

Most participants were on one type of medicine despite poor control. For many hypertensive patients, combination therapy may be required to achieve optimum control and combination therapy is believed to achieve better control than monotherapy as some drugs work synergistically as found in a study conducted in China [23]. This may be due shortage of doctors especially in rural areas as hypertensive medications can only be initiated or intensified by a doctor. The availability of other types of medicines may also be a challenge thus forcing the prescribers to continue with monotherapy even when it is inadequate. For example in this study, patients who were taking hydrochlorothiazide, the commonest medicine among the participants, had medicine was available throughout the previous year and were getting the medicine for free unlike other patients who were taking other medicines such as Nifedipine and Captopril who occasionally failed to get their medicines from the health facilities due to stock-outs.

Participants on a single dose regimen were less likely to have uncontrolled hypertension possibly because they were more compliant with their medications. This is an important although not surprising finding as previous studies have also revealed the same finding [23, 24]. Anti-hypertensive medications that are taken once daily are taken more regularly than drugs that have to be taken more than once daily [24]. According to Benson and Britten, since hypertensive medications are taken daily for life, a simple dosage is expected to reduce frustration and a propensity for non-compliance [25].

Use of traditional herbs for the management of hypertension was found to be generally high among the study participants as was reported in another study on hypertension control among black peri-urban South Africans [14]. The use of the herbs was also associated with having uncontrolled hypertension. The herbs being used may have a hypertensive effect rather than the intended anti-hypertensive effect and those taking the herbs may stop taking their prescribed anti-hypertensive medication resulting in elevated blood pressure.

Patient education is critical in the management of hypertension as was also demonstrated by this study. Patients who have been educated (by doctors or nurses) and understand their disease process and the consequences of inadequate blood pressure control tend to be better controlled. The low levels of perception of risk of developing complications due to hypertension could be due health workers not spending adequate time counseling patients because of possible staff shortages against a huge workload at the health facilities.

Availability of medicines at the health facilities was also found to be a challenge among participants, particularly those on the more expensive medicines such as the calcium channel blockers and ACE inhibitors. This could be a reflection of the financial constraints in the health system as a whole. Most participants were unemployed with low average household incomes and as a result when they failed to get medicine from the health facilities for free or at a subsidized rate they just waited until the medicines were available. Medicines for hypertension should be taken on a daily basis and unavailability of medicines for this population will breed non-compliance which has grave implications on hypertension control.

## Conclusion

Uncontrolled hypertension among hypertensive patients on treatment in Lupane district is a significant public health problem and interventions at the patient, the provider and the health delivery system are needed to improve hypertension control. The health benefits of weight reduction and exercise, reducing salt intake and compliance with medication need to be communicated to the hypertensive patients using appropriately tailored health education messages. There is need to prioritize NCDs such as hypertension and ensure availability of all the common anti-hypertensive medicines at all the health facilities in the district.

### Study limitations

The minimum sample size of 384 participants could not be reached because of limitations in time. The temporal relationship of some questionnaire responses and uncontrolled hypertension could not be assessed since this was a cross-sectional study. For example it cannot be concluded from this study that those who added salt to their food at the table started the habit before their blood pressure was uncontrolled or vice-versa.
